# Extracellular vesicles released from the filarial parasite *Brugia malayi* downregulate the host mTOR pathway

**DOI:** 10.1371/journal.pntd.0008884

**Published:** 2021-01-07

**Authors:** Alessandra Ricciardi, Sasisekhar Bennuru, Sameha Tariq, Sukhbir Kaur, Weiwei Wu, Abdel G. Elkahloun, Anush Arakelyan, Jahangheer Shaik, David W. Dorward, Thomas B. Nutman, Roshanak Tolouei Semnani

**Affiliations:** 1 Laboratory of Parasitic Diseases, National Institute of Allergy and Infectious Diseases, NIH, Bethesda, Maryland, United States of America; 2 Laboratory of Pathology, Center for Cancer Research, National Cancer Institute, NIH, Bethesda, Maryland, United States of America; 3 Microarray Core, Cancer Genetics and Comparative Genomics Branch, National Human Genome Research Institute, NIH, Bethesda, Maryland, United States of America; 4 Section of Intracellular Interactions, Eunice Kennedy Shriver National Institute of Child Health and Human Development, NIH, Bethesda, Maryland, United States of America; 5 RML Microscopy Unit, RML Research Technologies Section, National Institute of Allergy and Infectious Diseases, NIH, Hamilton, Montana, United States of America; University of Liverpool, UNITED KINGDOM

## Abstract

We have previously shown that the microfilarial (mf) stage of *Brugia malayi* can inhibit the mammalian target of rapamycin (mTOR; a conserved serine/threonine kinase critical for immune regulation and cellular growth) in human dendritic cells (DC) and we have proposed that this mTOR inhibition is associated with the DC dysfunction seen in filarial infections. Extracellular vesicles (EVs) contain many proteins and nucleic acids including microRNAs (miRNAs) that might affect a variety of intracellular pathways. Thus, EVs secreted from mf may elucidate the mechanism by which the parasite is able to modulate the host immune response during infection. EVs, purified from mf of *Brugia malayi* and confirmed by size through nanoparticle tracking analysis, were assessed by miRNA microarrays (accession number GSE157226) and shown to be enriched (>2-fold, p-value<0.05, FDR = 0.05) for miR100, miR71, miR34, and miR7. The microarray analysis compared mf-derived EVs and mf supernatant. After confirming their presence in EVs using qPCR for these miRNA targets, web-based target predictions (using MIRPathv3, TarBAse and MicroT-CD) predicted that miR100 targeted mTOR and its downstream regulatory protein 4E-BP1. Our previous data with live parasites demonstrated that mf downregulate the phosphorylation of mTOR and its downstream effectors. Additionally, our proteomic analysis of the mf-derived EVs revealed the presence of proteins commonly found in these vesicles (data are available via ProteomeXchange with identifier PXD021844). We confirmed internalization of mf-derived EVs by human DCs and monocytes using confocal microscopy and flow cytometry, and further demonstrated through flow cytometry, that mf-derived EVs downregulate the phosphorylation of mTOR in human monocytes (THP-1 cells) to the same degree that rapamycin (a known mTOR inhibitor) does. Our data collectively suggest that mf release EVs that interact with host cells, such as DC, to modulate host responses.

## Author summary

Lymphatic filariasis, a neglected tropical disease caused by parasitic worms which affects millions of individuals, represents an important public health concern due to its high morbidity that significantly diminishes quality of life. The parasite is able to establish a chronic infection by manipulating its host’s immune responses. The larval mf stage of the parasite is of particular interest as the parasites are present in the peripheral blood and in constant contact with the host’s immune cells. We decided to investigate the role of mf-derived EVs in the modulation of human antigen presenting cells during infection. We showed that mf release EVs that are similar to exosomes, and these parasite vesicles are readily internalized by human DC. The mf-derived EVs possess a unique miRNA profile and are enriched in miRNAs that can target the mTOR pathway. We have also demonstrated that purified mf-derived EVs are capable of inhibiting mTOR signaling in human monocytes. Collectively, our results suggest that mf release exosome-like vesicles that modulate the immune metabolism of host antigen presenting cells.

## Introduction

Spanning 73 countries, lymphatic filariasis affects more than 120 million people in poorer, rural tropical and subtropical areas [1]. Lymphatic filariasis, a neglected tropical disease caused by parasitic nematodes, represents an important public health concern due to its high morbidity that significantly diminishes quality of life [2].

Immune modulation and hyporesponsiveness are hallmarks of filarial infections. Chronic infections are characterized by elevated frequencies of IL-10 producing CD4^+^ T cells [3]. This shift toward a regulatory cytokine environment leads to the suppression of T cell proliferation and decreased IFN-γ and IL-2 production in response to filarial antigens as well as to bystander antigens [4–9]. We have previously demonstrated that the microfilariae (mf), the larval stage of the parasite, actively modulate the function of antigen presenting cells [10–15]. Mf induce cell death in dendritic cells (DC), while also inhibiting cytokine production and their ability to activate T cells [11,15]. Furthermore, we have shown that one way by which mf interfere with DC function involves the alteration of TLR expression [14].

The mechanistic target of rapamycin (mTOR) pathway is critical in regulating cellular growth, proliferation, and metabolism [16]. mTOR, a highly conserved serine/threonine protein kinase, makes up the catalytic subunit of two multiprotein complexes; mTOR complex 1 (mTORC1) and mTOR complex 2 (mTORC2) [17]. These two complexes regulate different cellular processes in response to various stimuli. Environmental signals activating the mTOR pathway include nutrients, hormones, and growth factors. mTORC1 regulates mRNA synthesis and protein translation as two of its main downstream targets include ribosomal subunit p70S6 kinase 1 (S6K) and eukaryotic initiation factor 4E-binding protein (4E-BP) [18]. Furthermore, mTORC1 induces cellular growth by promoting *de novo* lipid synthesis, glycolysis, and purine synthesis [19–21]. Along with promoting anabolism, the mTOR pathway also balances catabolism by regulating autophagy [16]. Signaling through the mTOR pathway is directly inhibited by the drug rapamycin. In the context of filarial infections, our previous work has shown that mf from *Brugia malayi* inhibit the mTOR pathway in human DC by downregulating the phosphorylation of mTOR itself [22]. We have also demonstrated that mf inhibit the phosphorylation of the downstream targets p70S6K and 4EBP1 [22]. These data suggest that extracellular helminth parasites downregulate mTOR signaling in human DC. To elucidate the mechanisms mediating this modulation, we focused on the parasite’s production of soluble factors, namely EVs.

EVs are secreted by most or all organisms, and they represent a mechanism of cell-to-cell communication. In the last decade or so, EVs have been acknowledged as an important component of parasite secretion products [23]. EVs allow for the packaging and protection of parasite cargo, such as proteins, genetic material, and lipids, that can then be taken up by other cells. Distinct EV types may be differentiated based on their biogenesis. For example, EVs derived from the endocytic pathway are called exosomes whereas those derived from shedding of the plasma membrane are known as microvesicles [24]. Exosomes are typically between 40-100nm in size and express markers associated with their biogenesis which involves inward budding of multivesicular bodies [23]. In contrast, microvesicles can be up to 1000nm in size and contain plasma membrane lipids and surface proteins [23,25]. However, it is also important to note that the field is now starting to move away from a strict classification of EV types based on size, as there are exceptions. In the context of parasitic infections, both protozoan and helminth parasites have been shown to use EVs to manipulate their hosts as well as to communicate within their environment. *Leishmania* species as well as *Trypanosoma cruzi* release EVs that contain immunomodulatory molecules which induce the secretion of IL-10, thereby, inhibiting the host’s inflammatory response [26,27]. Along with immune modulation, EVs also mediate communication between parasites. For instance, more adherent strains of the extracellular protozoan *Trichomonas vaginalis* release EVs that induce better adherence of weaker strains [28]. Helminths have also been shown to use EVs to regulate the host immune response and prevent pathogen clearance. The gastrointestinal nematode *Heligmosomoides polygyrus* releases EVs that inhibit the activation of type 2 innate lymphoid cells and suppress the expression of the IL-33 receptor [29]. It has been previously shown that all life cycle stages of the filarial parasite *B*. *malayi* release exosome-like vesicles [30].

In the present communication, we demonstrate that *B*. *malayi* mf release EVs that are similar to exosomes in size as well as appearance, and these parasite vesicles are internalized by human DC. The mf-derived EVs are not only enriched in microRNA that can target the mTOR pathway, but also significantly reduce the phosphorylation of mTOR in human monocytes. Together, our data suggest that *B*. *malayi* mf release exosome-like vesicles that modulate the metabolism of host antigen presenting cells by downregulating the mTOR downstream signaling.

## Methods

### Ethics statement

Human monocytes used for this study were isolated from leukopaks from healthy donors by counterflow centrifugal elutriation. This work was performed under Institutional Review Board (IRB)-approved protocols from the Department of Transfusion Medicine (Clinical Center, National Institutes of Health, Bethesda, MD). Furthermore, all donors provided informed written consent.

### *Brugia malayi* microfilariae maintenance and production of extracellular vesicles

Live *B*. *malayi* mf were provided by the FR3 at the University of Georgia, Athens, GA by peritoneal lavage of infected jirds and were purified as previously described [22]. The mf numbers were determined by microscopy and EV production was initiated by incubating up to 10 x 10^6^ parasites in 5 mL of mf media (RPMI 1640, 1% D-glucose, 1% L-glutamine, 1% penicillin/streptomycin) for 24 hours at 37°C in 5% CO_2_.

### Extracellular vesicle isolation and tracking

The ExoQuick-TC ULTRA kit (System Biosciences, Palo Alto, CA) was used, according to the manufacturer’s instructions, in order to isolate mf-derived EVs after 24 hours of incubation. A NanoSight NS300 and NTA software were used to assess the average size and concentration of the particles in the purified preparation [31].

### Proteomic analysis

The proteomic analysis of the purified EV samples was performed by Bioproximity, a mass spectrometry and proteomic service company (Manassas, VA). We sent Bioproximity four purified EV samples, each containing 2μg of protein. The four samples were from independent mf shipments. Purified mf-derived EV preparations were reconstituted in 5% SDS, 50 mM Tris-HCl, pH 8.0, 5 mM TCEP, 20 mM CAA and heated to 100°C, 10 min. Samples were cooled, probe sonicated and centrifuged to clarify. Samples were digested with trypsin using the SP3 method as previously described [32]. Bead eluates were vacuum centrifuged to dryness and resuspended in mobile phase A (97% MilliQ water, 2% acetonitrile, 0.1% formic acid).

Each digestion mixture was analyzed by UHPLC-MS/MS. Liquid chromatography (LC) was performed on an Easy-nLC 1200 UHPLC system (Thermo) as previously described [33]. MGF files were generated from mzML using OpenMS [34].

All searches were performed using the PEAKS 7 studio (version 7, Bioinformatics Solutions Inc) using a combined database of *B*. *malayi* and human proteins and decoy sequences containing both forward and reverse sequences as well as a common contaminant database (https://www.thegpm.org/crap/) using default parameters. Dynamic modifications of methionine oxidation and N-terminal acetylation as well as fixed modification of carbamidomethyl cysteine were included in the database search. Only tryptic peptides with up to two missed cleavage sites with a minimum peptide length of six amino acids were allowed. The false discovery rate (FDR) was set to 0.01 and threshold-based filtering of -10logP scores of 30 for both peptide and protein identifications.

### EV uptake assessement by flow cytometry

Parasite EVs were labeled using the Exo-Red exosome RNA fluorescent label kit (System Biosciences). A 180μg protein sample of EVs was incubated for 30 minutes at 37°C with 50μl of Exo-Red dye. Exo-spin columns were used to clean the EV stained preparation and remove excess dye (Cell Guidance Systems, St. Louis MO). 44μl of the stained EV preparation was added to 1x10^6^ THP-1 cells in 1mL of cell culture media (RPMI 1640, 10% FBS, 1% HEPES, 1% L-glutamine, 1% penicillin/streptomycin, 1% Sodium pyruvate, 1% Sodium bicarbonate). The cells and EVs were incubated for 10 minutes, 60 minutes, or 24 hours. At the end of the incubation, the cells were immediately washed with excess volume of Stain Buffer (FBS) (BD Biosciences, San Jose CA). The wash step was repeated three times. The cells were then resuspended in Stain Buffer (FBS) and analyzed on a BD LSRFortessa using BD FACSDiva Software. The acquired data was then analyzed using FlowJo software. The assay was repeated at least once.

### Confocal microscopy

Human monocyte derived dendritic cells were used to perform confocal microscopy assays. To generate dendritic cells, elutriated human monocytes were cultured at 37°C where recombinant human interleukin-4 and recombinant human granulocyte-macrophage colony-stimulating factor (Peprotech, Rocky Hill, NJ) were added at 50ng/ml on days 0, 3, and 6 of culture.

Mf-derived EV uptake by human dendritic cells was measured using confocal microscopy with Zeiss 780. Human monocyte-derived dendritic cells were labeled with PKH26 (red) and the mf-derived EVs were labeled with PKH67 (green) according to the manufacturer’s instructions (Millipore Sigma, Burlington, MA). Immediately following staining, the EVs were passed through Exo-spin columns to remove the dye. The EVs were then eluted using 1X PBS [35]. VECTASHIELD Hardset Antifade Mounting Media with DAPI was also used following the manufacturer’s instructions (Vector Laboratories, Burlingame, CA). Human dendritic cells were co-cultured with parasite EVs for 3 days. The co-culture was performed in an 8-well chambered coverglass (ThermoFisher) using 5000 cells/well and 2.2 x 10^6^ stained EVs. Settings for the acquisition of confocal images were as previously described [35].

### Microarray analysis of miRNA

Mf supernatant and EV miRNA was isolated using MirVana PARIS kit (ThermoFisher Scientific). Three biological replicates were performed across different batches of EV. Each replicate consisted of 1.1 x 10^6^ mf. The supernatants used were not EV depleted. The concentration and RNA quality was measured using RNA-Bioanalyzer 2100 (Agilent, Santa Clara, CA). We used 200ng of miRNA from each replicate for labeling with the FlashTag™ Biotin HSR RNA Labeling Kit (Affymetrix-ThermoFisher Scientific, cat#901911, Walthman, MA). The labelled RNAs were then hybridized overnight on the Affymetrix GeneChip miRNA arrays 4.0. All washing, staining and scanning steps were done according to the manufacturer’s instructions. The.cel files were imported into JMP Genomics Suite (SAS Institute), RMA normalized and ANOVA analysis was performed.

The differential analysis was performed by finding differentially expressed genes between mf EV and supernatant and using Limma with FDR of 0.05. The differentially expressed miRNAs were queried against mirDB to find target genes with interaction scores greater than 90.

The Genechip miRNA 4.0 Affymetrix arrays were imported into TACX (Transcriptome Analysis Console, Applied Biosystems) and analyzed with default settings of the miRNA 4.0.tac configuration file. The summary of the results is based on the RMA-DABG algorithm of TACX. The Robust Multi-chip Analysis takes into consideration background adjustment, quantile normalization, and summarization. The presence or absence of a miRNA species is based on the criteria of a probe set being detected in at least 50% of the samples above the background signal (DABG: Detected Above Background). The array comprises miRNA probes from different organisms. *Brugia* specific miRNAs in the EVs can be missed in this approach and would require a large sequencing approach. In this approach only well-known miRNAs from *B*. *malayi* and miRNAs that are highly similar with other organisms can be identified. Thus, we can expect to see multiple hits for the same miRNA from multiple organisms. Hence, we also grouped them all as unique miRNAs. The microarray data have been uploaded to the Gene Expression Omnibus at NCBI with the accession number GSE157226.

The target genes were then presented as input to find enriched pathways using ingenuity pathway analysis toolkit. The mTOR pathway was selected to display interactions between miRNA and their targets.

### RNA isolation and RT-PCR

A 100ul sample of purified EVs was used to extract enriched small RNA using the Total Exosome RNA and Protein Isolation Kit (Thermo Fisher Scientific) following the manufacturer’s instructions. Using the Taqman Advanced miRNA cDNA Synthesis Kit (Thermo Fisher Scientific) cDNA was made according to manufacturer’s instructions. 1–10 ng of miRNA extracted from purified EVs was used to perform polyadenylation tailing reaction at 37°C for 45 minutes and then 65°C for 15 minutes. This was followed by adenylation ligation reaction that was performed at 16°C for 60 minutes. The reverse transcriptase reaction was performed at 42°C for 15 minutes followed by 85°C for 5 minutes. An amplification step was performed at 95°C for 5 minutes for 1 cycle, 95°C for 3 seconds for 14 cycles, 60°C for 30 seconds for 14 cycles, and 99°C for 10 minutes for 1 cycle. RT-PCR was performed in order to screen the EV extracts for different microRNA. The reaction mix contained 18ul of cDNA, 2 ul of each respective primer and 20ul of Taqman Advanced Mastermix. Cycling parameters were 95°C for 20 seconds for 1 cycle, 95°C for 40 cycles and 60°C for 40 cycles. A Ct value of 40 is considered to be negative. All of the primers used were purchased commercially: let-7 (ThermoFisher Scientific, catalog # A25576, ID 778575_mir), mir-100 (ThermoFisher Scientific, catalog # A25576, ID 478224_mir), mir-155 (ThermoFisher Scientific, catalog # A25576, ID 483064_mir), mir-7 (ThermoFisher Scientific, catalog # 4427975, ID 002555), and mir-71 (ThermoFisher Scientific, catalog # 4440886, ID 001364).

### pmTOR flow cytometry

THP-1 cells were maintained in culture at a concentration not exceeding 1x10^6^ cells/ml. For stimulation experiments, 1x10^6^ THP-1 cells were aliquoted in different tubes in a final volume of 1ml serum-free media. The cells were treated with different stimuli and incubated for 60 minutes at 37°C. The different stimulation conditions included an unstimulated control (media alone), 0.1μM rapamycin, 50,000 live mf, and mf-derived EVs normalized as 10ug of protein content. At the end of the stimulation period, the cells were immediately fixed with equal volume of IC Fixation Buffer (eBioscience, Thermo Fisher Scientific), vortexed, and incubated for 40 minutes at room temperature protected from light. The cells were then centrifuged at 600xg for 7 minutes, and the supernatant was discarded. The cell pellets were resuspended in the residual volume, and 1ml of ice-cold 100% methanol was added. The samples were vortexed and incubated for 30 minutes at 4°C. The cells were washed with an excess volume of flow cytometry staining buffer (PBS with 1%FBS) and centrifuged at 600xg for 7 minutes. The supernatant was discarded, and 10μl of human Ig was added to each sample and incubated for 10 minutes at room temperature. Phospho-mTOR (Ser2448) monoclonal antibody (MRRBY)-PE (eBioscience, Thermo Fisher Scientific) was added to each tube at 2.5ng/test. The volume for each tube was brought to 100μl using flow cytometry staining buffer, and the samples were incubated for 60 minutes at room temperature protected from light. Then, 3 mL of flow cytometry staining buffer was added to each sample. They were centrifuged at 600xg for 7 minutes, and the supernatant discarded. This wash step was repeated one time. The stained cells were resuspended in 200 μl of flow cytometry staining buffer. The samples were analyzed on a BD LSRFortessa using BD FACSDiva Software. The acquired data was then analyzed using FlowJo software.

### Statistical analysis

For analysis of flow cytometry data, Wilcoxon test was used in order to compare nonparametric paired data. These statistical analyses were performed using GraphPad Prism 7 (La Jolla, CA). *P* values less than 0.05 were considered significant.

## Results

### Mf release exosome like vesicles

**Fig 1:**
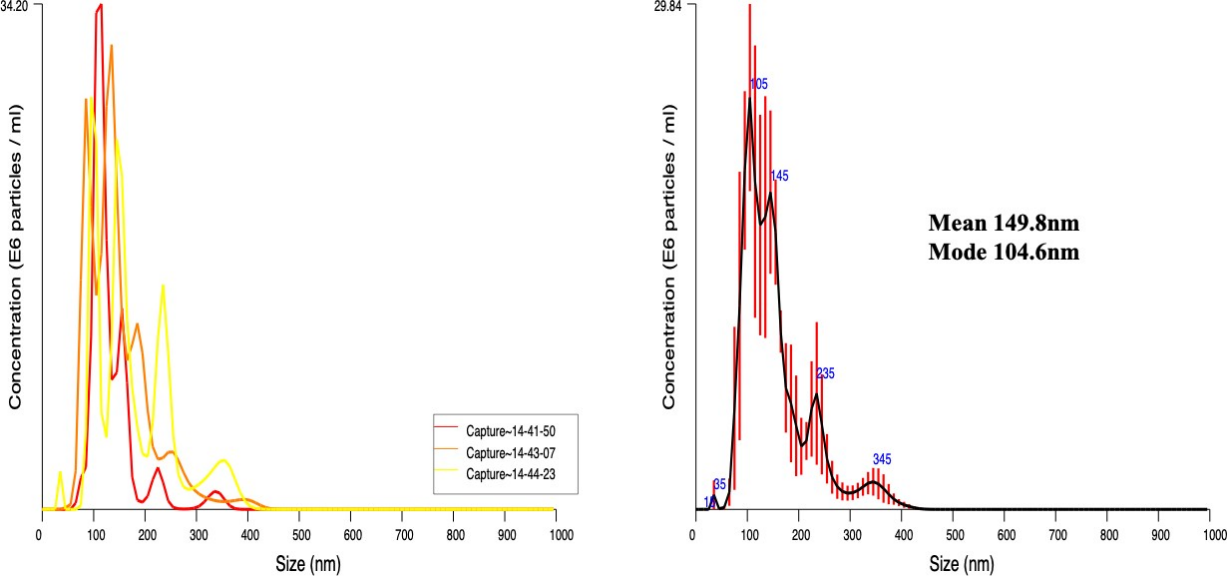
Characterization of mf-derived EVs. Live *B*. *malayi* mf were cultured for 24 hours at 37°C in 5% CO_2_. EVs were then purified from the mf culture media. The profile of these isolated EVs was assessed using a NanoSight NS300 and NTA software. The majority of the EVs in the sample fall within the size range of exosomes.

**Fig 2:**
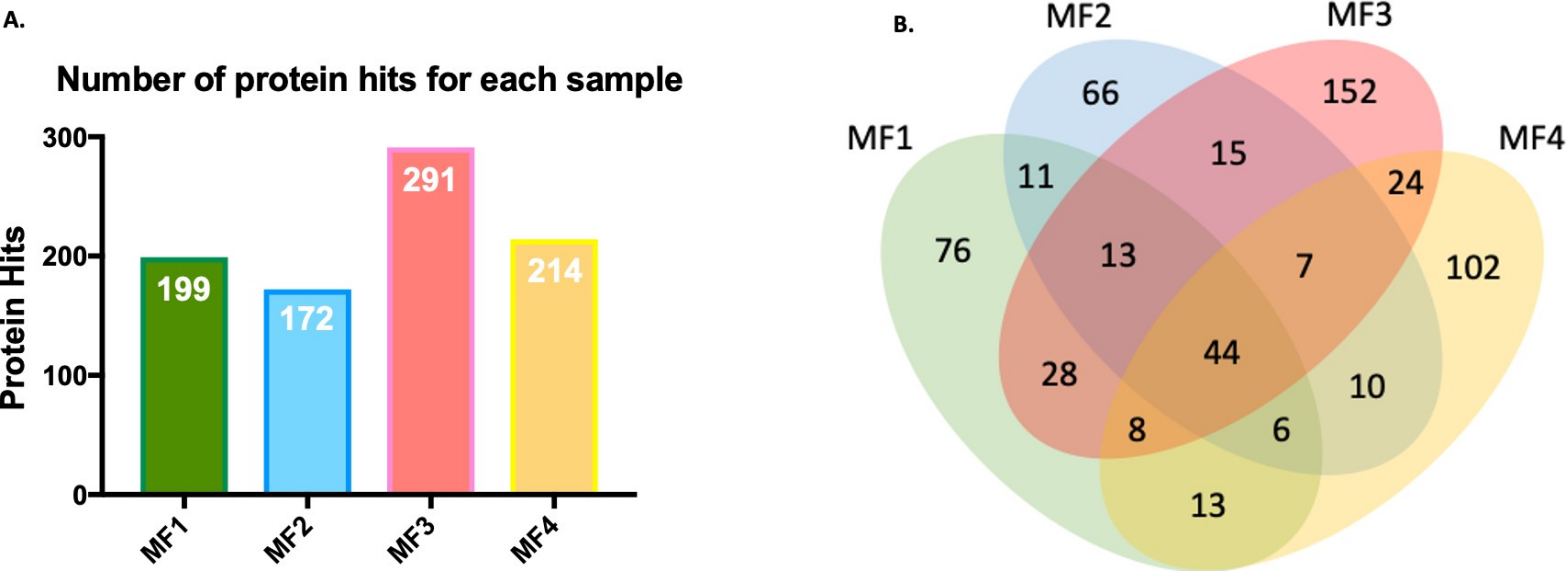
Proteomic characterization of mf-derived EVs. Four mf-derived EV samples were prepared for liquid chromatography- tandem mass spectrometry. The proteomic analysis revealed several parasite proteins. The total number of identified filarial proteins differed slightly between the EV preparations (A). Proteomics results for each EV sample was assessed in order to determine overlapping protein hits (B).

Mf were cultured for 24 hours in order to allow EV release. Using the parasite culture media, we isolated and purified vesicle samples. The size of these EV preparations was assessed by nanoparticle tracking analysis. From three separate captures, the NS300 NTA software calculated a particle size mean of 149.8 nm and a mode of 104.6 nm (Fig 1). Although the majority of the EVs in the sample were approximately 100 nm in size, the analysis noted the presence of a small population of EVs with a size range of 235 nm-345 nm (Fig 1). Particles that are 100 nm or smaller fall in the size range of exosome [23]. Thus, a large portion of our preparation consists of EVs that are exosome-like. As previously mentioned, our tracking analysis also identified a subset of larger EVs which are most likely microvesicles [23], suggesting the isolation of heterogenous populations of EVs. Our EV preparations were further analyzed by liquid-chromatography tandem mass spectrometry in order to characterize the protein cargo of the vesicles (Table 1 and Fig 2). Four separate mf EV preparations were analyzed (Fig 2A), and 44 filarial proteins were detected to be present in all preparations (Fig 2B). The proteomics analysis revealed that our mf-derived EVs contained elongation factor 1-α which is a characteristic marker of mammalian exosomes [36], histones, heat shock proteins, and ATP synthase which have all been reported in exosomes derived from various sources [36–39] (Table 1).

**Table 1:** Proteins present in all EV preparations analyzed.

Accession Number	Protein Description
P90689|ACT_BRUMA	Actin
A0A0K0JJB8_BRUMA	Actin
A0A1P6C3D2_BRUMA	Bm5086 (Actin)
A0A0H5SER1_BRUMA	Bm8524 isoform a (Actin)
P27541|HSP70_BRUMA	Heat shock 70 kDa protein
A0A0K0JHW8_BRUMA	Elongation factor 1-alpha
A0A0H5S3P5_BRUMA	Phosphoglycerate kinase
A0A0H5S7Y7_BRUMA	BMA-DAF-21 (HSP-90)
A0A0K0K0E4_BRUMA	Histone H2A
A0A0K0J213_BRUMA	Bm1715 (vWA)
A0A0K0IZ73_BRUMA	ATP synthase subunit alpha
A0A0J9XXG7_BRUMA	Glyceraldehyde-3-phosphate dehydrogenase
A0A1P6C609_BRUMA	BMA-ENPL-1 isoform a
A0A0K0IZQ6_BRUMA	Bm13965 (bma-enol-1)
A0A0K0IZ71_BRUMA	ATP synthase subunit beta
A0A0I9R327_BRUMA	Eukaryotic translation initiation factor 5A
A0A1P6C045_BRUMA	Histone H4
A0A0K0IYR7_BRUMA	BMA-TUFM-1
A0A0K0IRL6_BRUMA	Bm8805
A0A158Q277_BRUMA	Uncharacterized protein
A0A158Q275_BRUMA	Uncharacterized protein
A0A158Q274_BRUMA	Uncharacterized protein
A0A158Q276_BRUMA	Uncharacterized protein
A0A0K0IYG8_BRUMA	Bm13661 (HSP-70)
A0A0K0J5L7_BRUMA	Bm2363 isoform e
A0A0K0J5L8_BRUMA	Bm2363 isoform e
A0A0K0J5L6_BRUMA	Bm2363 isoform e
A0A0K0JSW0_BRUMA	Uncharacterized protein
A0A0K0JD75_BRUMA	Histone H3
A0A0K0JRX5_BRUMA	Uncharacterized protein
A0A0K0JJA2_BRUMA	BMA-TAG-189
A0A0R3REA3_BRUMA	Uncharacterized protein
A0A0R3REA2_BRUMA	Uncharacterized protein
A0A158T1L3_BRUMA	BMA-NMTN-1 isoform a
A0A0K0JED9_BRUMA	Bm4375 isoform a
A0A0J9Y2D8_BRUMA	Bm4375 isoform a
A0A0K0JED8_BRUMA	Bm4375 isoform a
A0A0K0JED5_BRUMA	Bm4375 isoform a
A0A0H5SBL8_BRUMA	Bm4375 isoform a
A0A158PTJ7_BRUMA	Bm6177 isoform f
A0A0K0ISD1_BRUMA	Uncharacterized protein
A0A0J9XXN3_BRUMA	Bm7266
A0A0K0IVY8_BRUMA	Bm12835
A0A0I9NBR2_BRUMA	Bm12804

Four mf-derived EV samples were prepared for liquid chromatography- tandem mass spectrometry. The proteomic analysis revealed several parasite proteins. This table contains the 44 proteins that were detected in all 4 EV preparations.

### Human dendritic cells internalize mf-derived EVs

**Fig 3:**
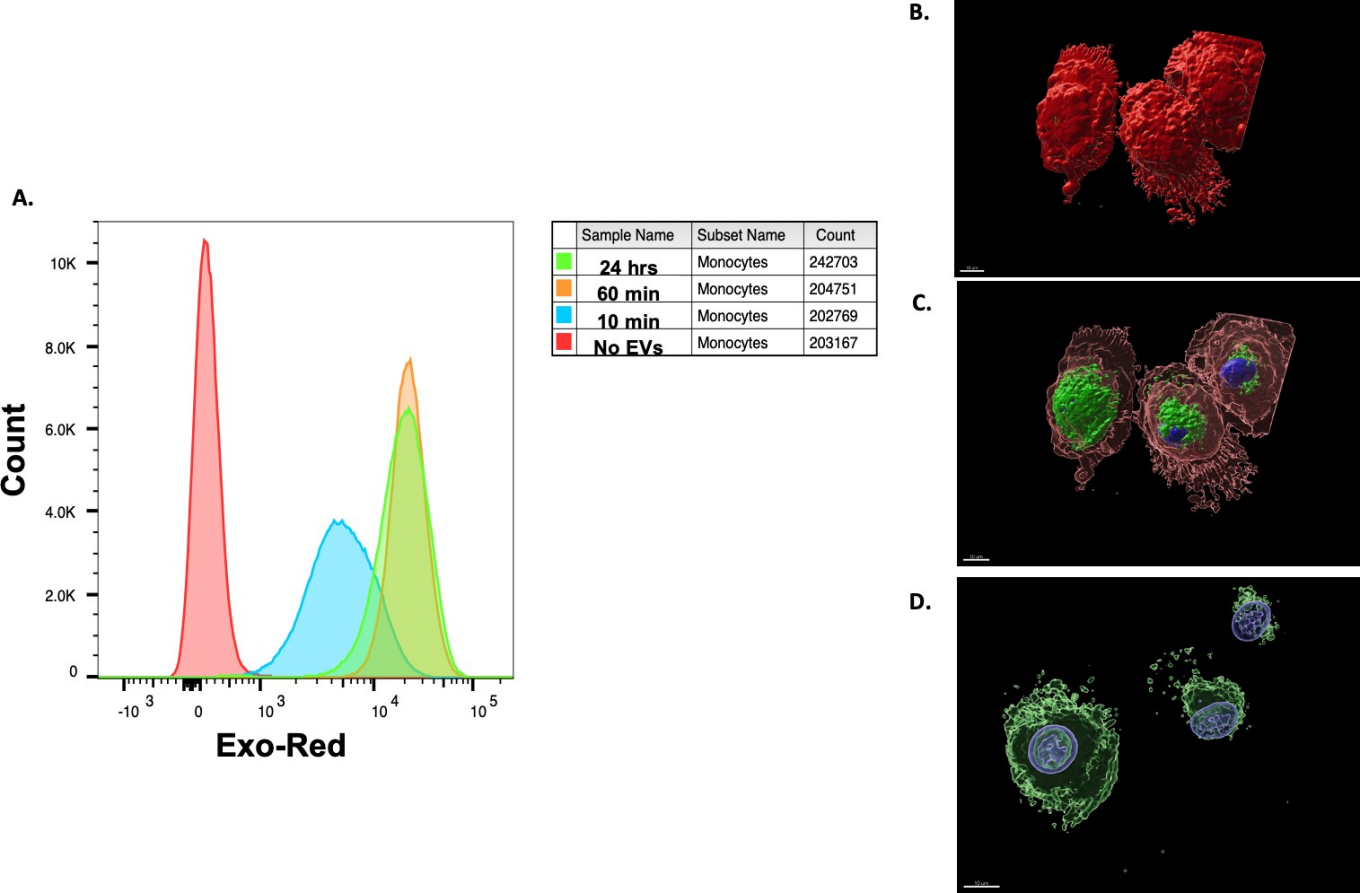
Internalization of mf-derived EVs by human DC. THP-1 cells were incubated for 10 minutes, 60 minutes, or 24 hours with mf-derived EVS that were stained with Exo-Red dye. EV uptake was assessed by flow cytometry (A). Human monocyte derived dendritic cells were co-cultured with *B*. *malayi* mf-derived EVs for 72 hours at 37°C in 5% CO_2_. The dendritic cells were labelled with PKH26 (red) and counterstained with VECTASHIELD Hardset Antifade Mounting Media with DAPI (blue) to visualize the nuclei. The parasite EVs were labelled with PKH67 (green). The merged image demonstrates that no parasite-derived EVs remain bound on the surface of the cell (B). The internalized vesicles appear diffused throughout the cytoplasm of the cells (C), with some EV products potentially translocating into the nucleus (D). All images were captured using Zeiss 780. The assay was performed three times.

Our previous work has demonstrated that live mf modulate dendritic cell responses by inducing apoptosis, inhibiting their secretion of certain cytokines, and reducing their ability to activate CD4^+^ T cells [11, 15]. Through the use of uptake experiments assessed by flow cytometry, we showed that mf-derived EVs were readily internalized by human monocytes within 10 minutes of co-culture (geometric mean fluorescence intensity (geo MFI) of 5,796) (Fig 3A). We observed an increase in signal at 60 minutes (geo MFI of 21,119) that was maintained at 24 hours (geo MFI 17,911) post incubation. Cells with no mf-derived EVs were used to confirm negligible background fluorescence. Confocal images of fixed cells further confirmed the internalization of labeled mf-derived EVs, and that no EVs remained bound to the dendritic cell surface (Fig 3B and 3C, and S1 Video). Furthermore, our data show some mf-derived EV products diffused throughout the cytoplasm with some appearing in the nucleus of the dendritic cell as well (Fig 3D and S1 Video). Therefore, there is an interplay between host immune cells and EVs that are released by mf during infection.

### Mf-derived EVs have a distinctive nucleic acid profile

**Fig 4:**
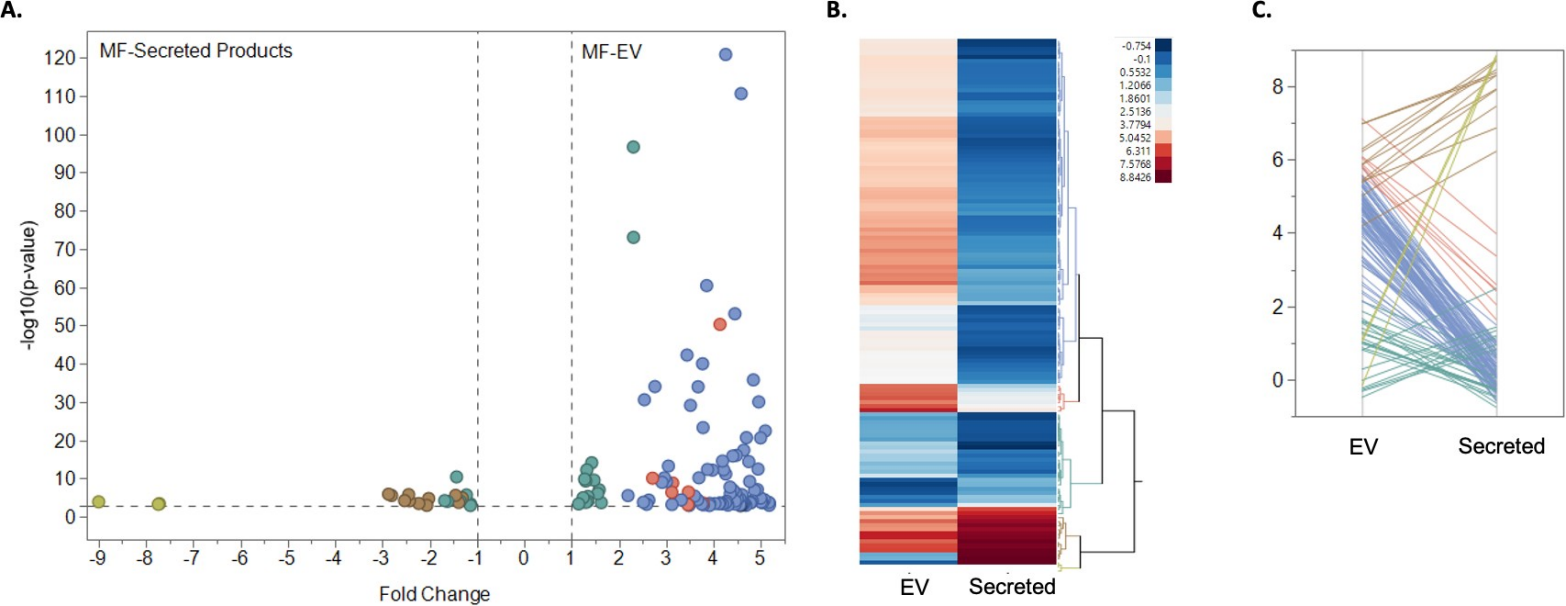
Overview of the differential expression of miRNAs between mf EVs and secreted products. Microarray analysis was used to assess differential miRNA expression between mf-derived EVs and mf secreted products. The miRNAs differentially expressed in mf secreted products were compared to those of mf EVs using log_2_-fold changes on the x-axis and adjusted *P* values (log_10_) on the y-axis (A). The heat map illustrates the hierarchical clustering of the normalized expression of miRNAs from mf EVs and secreted products with blue to red indicating low to high expression (B). The parallel coordinate plots of miRNAs demonstrate the downregulation or upregulation when comparing mf EVs and mf secreted product samples (C). Throughout the figures, brown/tan lines and dots represent downregulation in EVs, green lines and dots represent slight differential expression between EVs and secreted products, and blue/red lines and dots represent enrichment in EVs.

It is well known that EVs contain nucleic acids such as small non-coding RNAs in their cargo [23]. We were particularly interested in the miRNA signature of mf-derived EVs as this could provide insight into the vesicles’ potential modulatory functions. We performed a microarray analysis of RNA isolated from either mf-derived EVs or total mf secreted products. The purpose of this analysis was to examine whether parasite EV miRNA content differed significantly from what is detected in the supernatant. There were many miRNA that were equally present in both the EVs as well as the secreted products; however, the obtained data revealed 130 differentially expressed miRNA in the EVs compared to the secreted products. Since our microarray analysis yielded other organisms that also have the same miRNAs, we removed the prefix of the organism names and performed a count for all of the unique IDs, which could be broadly grouped into 60 different miRNA groups (Fig 4). Some miRNA were upregulated in the secreted products compared to the EVs (Fig 4). However, the majority of the differentially expressed miRNA were upregulated in the mf-derived EVs compared to the parasite secreted products (Fig 4). These significant differences in miRNA content depict the signature nucleic acid profile of EVs.

We next performed target gene analysis focusing on particular pathways that could be involved in the modulation of antigen presenting cells and their ability to drive an immune response. Our analysis showed that several miRNA present in EVs could target genes related to the mTOR signaling pathway (Table 2). These included mir-100, mir-7, mir-71, let-7, mir-99, mir-9, mir-34, mir-31, mir-92, and mir-4299.

**Table 2:** EV miRNAs with potential target genes in the mTOR pathway.

miRNAs present in mf-derived EVs	Target genes in mTOR pathway
mir-100 mir-99a mir-7	mTOR
mir-34a mir-9	Ras
mir-31 mir-4299 mir-92a	PI3K
Let-7	eIF-4E
mir-71	PDK1

Differentially expressed miRNAs identified through microarray analysis were queried against mirDB to find target genes with interaction scores greater than 90. The target genes were then presented as input to find enriched pathways using ingenuity pathway analysis toolkit. The mTOR pathway was selected to display interactions between miRNAs and their targets.

### Mf-derived EVs contain miRNAs that can target the mTOR pathway

**Fig 5:**
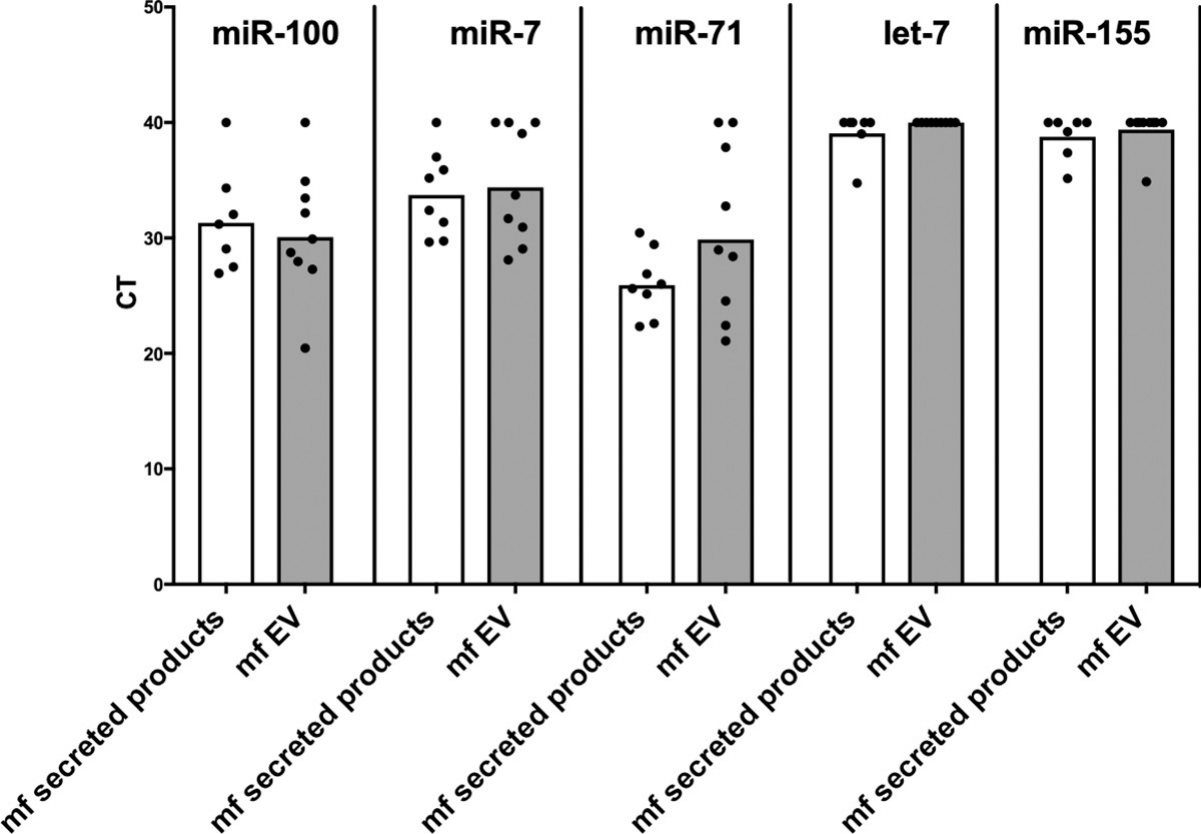
Presence of miRNAs predicted to target the mTOR pathway found in mf-derived EVs. cDNA was synthesized from small RNA that was extracted from mf-derived EVs. RT-PCR was then performed in order to screen EV nucleic acid cargo for miRNAs potentially targeting the mTOR pathway. The miRNA of interest included mir-100, mir-7, mir-71, let-7, and mir-155 as they are predicted to target genes relating to the mTOR pathway.

To expand on the information obtained by microarray analysis of miRNA present in the mf-derived EV cargo, we sought to confirm, by RT-PCR, the presence of certain miRNA known to target the mTOR pathway. In doing so, we assessed the miRNA content of mf-derived EVs. It was revealed that EVs derived from mf contained miR-100, miR-7, and miR-71 (Fig 5). The levels of these miRNA, known to target the mTOR pathway, were comparable between parasite secreted products and EVs. These data suggest that the main source of these specific miRNA found in the secreted products may, in fact, be EVs.

### Phosphorylation of mTOR in human monocytes is inhibited by mf-derived EVs

**Fig 6:**
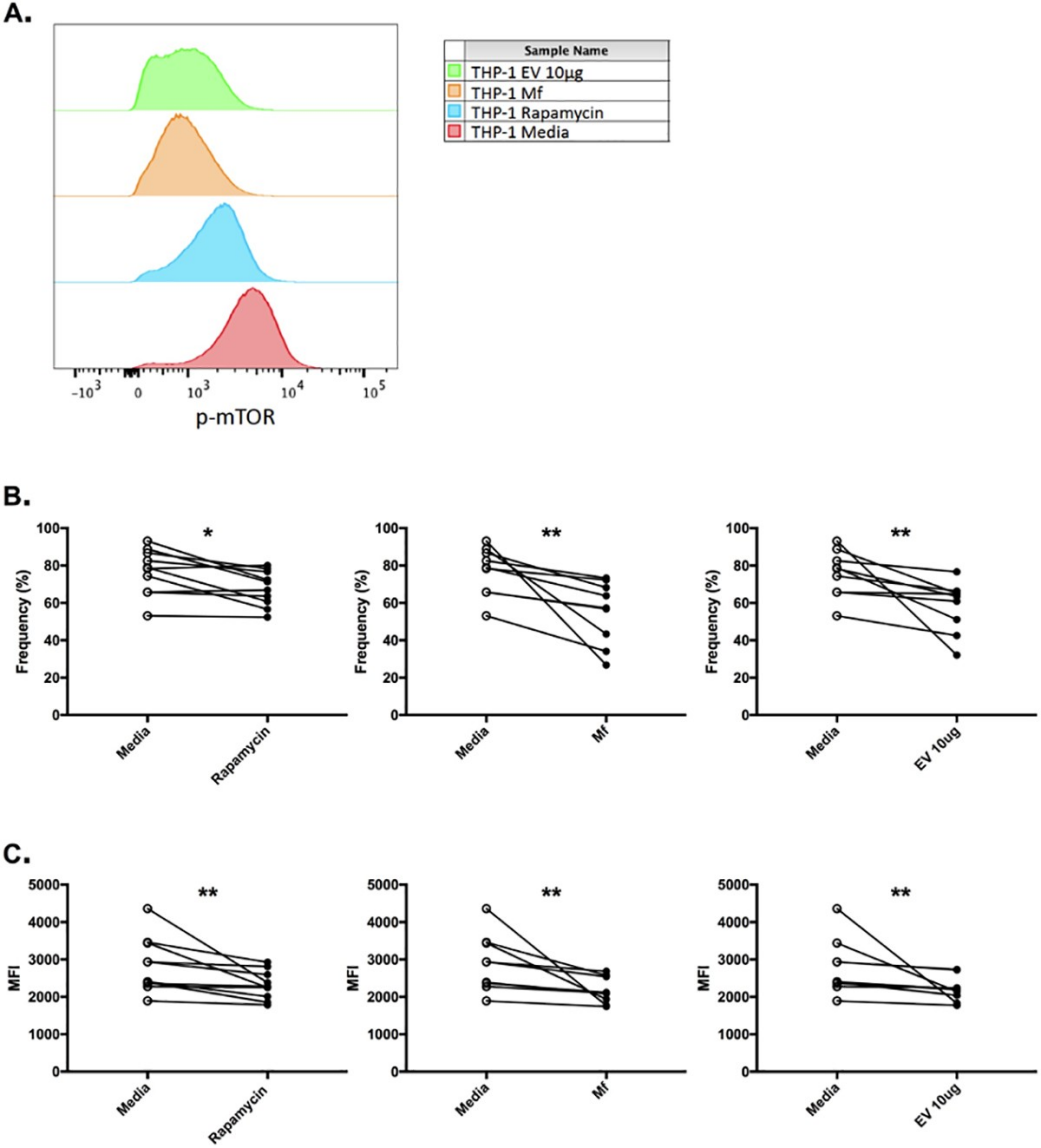
Mf-derived EVs downregulate mTOR phosphorylation in human monocytes. Human THP-1 cells were cultured in the presence of media alone, rapamycin, live mf, or mf-derived EVs for 1 hour at 37°C in 5% CO_2_. Following the stimulation, fixation, permeabilization, and intracellular staining for phosphorylated mTOR was performed on the cells. The samples were analyzed by flow cytometry; a representative plot demonstrates the assessment of phosphorylated mTOR (Phospho-mTOR (Ser2448)) (A). The results are presented as both frequencies (B) and MFI values (C). n = 10 for rapamycin condition, n = 9 for both live mf and EV conditions. *: ≤ 0.05, **: ≤ 0.01.

Previous work has demonstrated that live mf are capable of inhibiting the phosphorylation of mTOR in human antigen presenting cells [22]. In order to determine whether mf-produced EVs can downregulate mTOR, we evaluated the phosphorylation of mTOR in a human monocytic cell line by flow cytometry. Rapamycin and live mf were included in our assays as controls; the former being a known inhibitor of mTOR. As shown, the basal levels of phospho-mTOR are high in human monocytes, and, as expected, a statistically significant decrease in phosphorylation, assessed by frequency and geo MFI, was observed when rapamycin was added; p = 0.0195 and p = 0.0020, respectively (Fig 6B and 6C). The addition of rapamycin inhibited the frequency of phospho-mTOR by 11%. Incubating human monocytes with live mf resulted in significantly decreased mTOR phosphorylation compared to basal levels (p = 0.0039 for frequency and p = 0.0039 for geo MFI) (Fig 6B and 6C); thus, corroborating our previous findings [22]. Live parasites inhibited the frequency of phosphorylated mTOR by 27%. This downregulation of phospho-mTOR by live mf was greater than what was observed with rapamycin; indicating the parasite’s potent inhibitory potential. Next, we sought to determine whether the parasite’s effect on mTOR could be attributed to EVs. Our data suggest that in the presence of mf-derived EVs, mTOR phosphorylation, assessed by both frequency and geo MFI, was significantly decreased when compared to basal levels (p = 0.0039 and p = 0.0039, respectively); an effect similar to live mf or rapamycin (Fig 6B and 6C). Purified mf-derived EVs inhibited the frequency of phospho-mTOR by 21.5%. It is important to note that none of the conditions affected the viability of the cells. These findings collectively suggest an inhibitory role for EVs in the mTOR pathway of human antigen presenting cells.

## Discussion

Parasitic nematodes have evolved with humans, resulting in an intimate relationship between host and pathogen that is characterized by active immune modulation and developed tolerance which leads to established chronic infections capable of spanning decades. Within the last few years, EVs have grasped the attention of groups interested in host-parasite interactions. EVs, secreted by most organisms, represent a communication mechanism through which genetic information, immunomodulatory molecules, and virulence factors may be transferred between cells. In this current study, we focused on EVs released by the mf stage of the filarial parasite *B*. *malayi*. Immune modulation, particularly antigen presenting cell dysfunction, represents a hallmark of chronic filarial infections. In order to build upon this known phenomenon, we chose to investigate the potential contribution of EVs in parasite mediated immune effects.

The majority of EVs isolated from *B*. *malayi* mf were within the size range of exosomes [23]. A small fraction of the vesicles isolated from mf were slightly larger than the expected exosome size range; thus, likely making them more similar to microvesicles. Unlike mammalian exosomes that have been extensively defined, there are no agreed upon markers to confidently characterize filarial exosomes. For this reason, mf vesicles that resemble exosomes in size are simply referred to as exosome-like in order to remain cautious (Fig 1). However, our proteomic analysis of the mf-derived EVs revealed proteins that are commonly found in mammalian and parasitic exosomes (Fig 2). These proteins, which include elongation factor 1-alpha, histones, and heat shock proteins, are believed to be packaged as a result of the exosome biogenesis process; thereby, indicating greater exosome-like properties of our purified sample [36–39]. We observed some variation in the protein cargo among the EV samples from the different mf biological replicates. These *in vivo* changes in EV protein cargo may be linked to a myriad of biological causes such as nutrient availability, host source, and simply parasite fitness. In fact, this observation, further demonstrates the link between EVs and parasite biology.

As previously mentioned, a great deal of work in the past has demonstrated that live mf have a significant effect on human antigen presenting cell (APC) function, consequently, affecting the host’s T cell response to filarial infection as well as to bystander antigens [4–9]. As part of this immune modulation paradigm, parasite-derived EVs must be able to interact with human APCs (e.g. DCs). We have demonstrated that EVs released by mf are readily internalized by human monocytes within the first 10 minutes of co-culture (Fig 3A). EVs do not remain on the APC surface, as shown by confocal microscopy. In fact, they are quickly internalized and are visualized throughout the cell cytosol. Furthermore, confocal imaging suggests that the mf-derived EVs may also translocate into the cell nucleus (Fig 3D and S1 Video). There is an emerging family of plant as well as mammalian pathogens known as nucleomodulins that can deliver effector proteins to the host nucleus in order to modulate host responses and successfully establish infection [40,41]. These effects are carried out by interfering with transcription, chromatin remodeling, RNA splicing, or DNA replication [40]. In the case of plant cells, each main class of pathogenic organism, fungi, bacteria, viruses, nematodes, and oomycetes, has been identified as having factors capable of translocating to the nucleus [41]. For human pathogens, this has been observed with *Listeria*, *Chlamydia*, *Shigella*, and *Escherichia*. [42–45]. The potential nuclear transportation of EV products will need to be examined further in order to confirm such transportation, to determine whether the EVs are intact or degraded, and to elucidate the mechanism mediating such effects. The rapid internalization of parasite vesicles carrying potential immune modulatory molecules by human dendritic cells could be a mechanism by which the live parasites exert their potent effects on the host immune response.

In fact, the mf circulate throughout the host peripheral blood and are at a constant interface with host cells. In only 24 hours, mf can secrete approximately 1.9 x 10^4^ EVs per parasite [30]. An infected individual can have thousands of mf per milliliter of blood; therefore, the number of total EVs released in the peripheral blood can be substantial [46,47]. It is also known that live mf make contact with the surface of human dendritic cells [11,48]. Therefore, this close proximity in combination with the elevated number of parasite EVs being released represents an environment where host dendritic cells are exposed to a high local concentration of parasite-derived EVs. The mechanism of EV internalization as well as the processing that occurs within the human DC are still unclear. Understanding the different endocytic pathways that potentially mediate EV internalization and processing by DC could help elucidate how the parasite alters the host response during infection.

Along with carrying protein cargo, EVs also contain miRNA. Host miRNA are key mediators in regulating innate and adaptive immune pathways [49]. Pathogens have evolved mechanisms to subvert host miRNA, but they can also encode their own small non-coding RNAs that help promote infection [49]. For example, it has been demonstrated that *H*. *polygyrus* miRNAs are transported within human cells through EVs and can then target genes to suppress inflammation as well as innate responses [29]. We collected *B*. *malayi* mf secreted products and compared the miRNA content to that found in the purified mf-derived EVs. We demonstrated, by microarray analysis, that there were 130 differentially expressed miRNAs in mf-derived EVs as compared to the mf secreted products (Fig 4). These differentially expressed miRNA categorized into broadly 60 different miRNA groups with mir-100 being one of the most represented miRNA families. This observation indicates a completely diverse miRNA profile for mf-derived EVs compared to the parasite’s entire pool of secreted products. Taken together these results indicate that the nucleic acid content of parasite EVs is markedly different from what is observed from overall contents being secreted; thereby, creating a microenvironment potentially crafted to aid in the establishment of infection.

Previous work has demonstrated that live mf downregulate the mTOR signaling pathway by inhibiting the phosphorylation of mTOR itself as well as its downstream effectors 4EBP1 and p70S6K [22]. The mTOR complex is a master regulator of immune metabolism as it mediates both catabolism and anabolism [16,19–21]. By inhibiting this pathway, mf can modulate the host cellular metabolism. We have also previously shown that this downregulation of mTOR results in increase autophagy [22]. As mentioned, we have demonstrated the effect of mf on the mTOR pathway in antigen presenting cells. However, the mechanism of action still required elucidation. We chose to focus on mf soluble secreted products, namely EVs. Mf-derived EVs contain their own miRNA signature. Notably, these vesicles contained mir-100, mir-7, and mir-71, which were all confirmed by microarray analysis as well as PCR (Fig 5). These miRNA can potentially target the mTOR pathway. Finally, the incubation of mf-derived EVs with human monocytes resulted in a significant decrease in the phosphorylation of mTOR (Fig 6). This inhibition of mTOR phosphorylation by the parasite EVs was as potent or greater than that observed with rapamycin, a known inhibitor of mTOR. Furthermore, the effect of EVs on the mTOR pathway within human monocytes recapitulated the effect observed with live mf. Thus, these observations suggest that the significant inhibitory effect exerted by mf on the mTOR pathway of antigen presenting cells is likely mediated in part by the release of EVs by the parasite. Additionally, the presence of miRNA targeting mTOR within mf-derived EVs may indicate one specific method used to downregulate the pathway.

Overall, the results of the presented study suggest that mf-derived EVs are significant contributors to the immune modulation driven by filarial infections. These vesicles are a part of the parasite’s secreted products. However, we have demonstrated that they contain particular miRNAs, potentially to carry out specialized functions. We have also confirmed the presence of several regulatory miRNAs within the mf-derived EVs that target genes belonging to the mTOR signaling cascade. EVs are readily taken up by host antigen presenting cells and can interfere with a major regulatory pathway by downregulating mTOR phosphorylation. Further research focusing on filarial EVs would expand the fields of both parasite biology and pathogen-host interactions by allowing for a greater understanding of the underlying mechanisms that govern parasite fitness as well as infection driven immune reprograming.

## Supporting information

S1 Video Internalization of mf-derived EVs by human DC. Human monocyte derived dendritic cells were co-incubated with *B*. *malayi* mf-derived EVs for 72 hours at 37°C in 5% CO_2_. The dendritic cells were labelled with PKH26 (red) and counterstained with VECTASHIELD Hardset Antifade Mounting Media with DAPI (blue) to visualize the nuclei. The parasite EVs were labelled with PKH67 (green). The video represents merged images allowing to visualize the different layers of cellular internalization. All images were captured using Zeiss 780. The assay was performed three times.
(PPTX)
