# Supplementary figures and images for: Extracellular vesicles released from the filarial parasite Brugia malayi downregulate the host mTOR pathway
Source: PLoS Negl Trop Dis. 2021 Jan 7;15(1):e0008884. doi: 10.1371/journal.pntd.0008884 (PMC7790274; doi:10.1371/journal.pntd.0008884)

## Slide 1
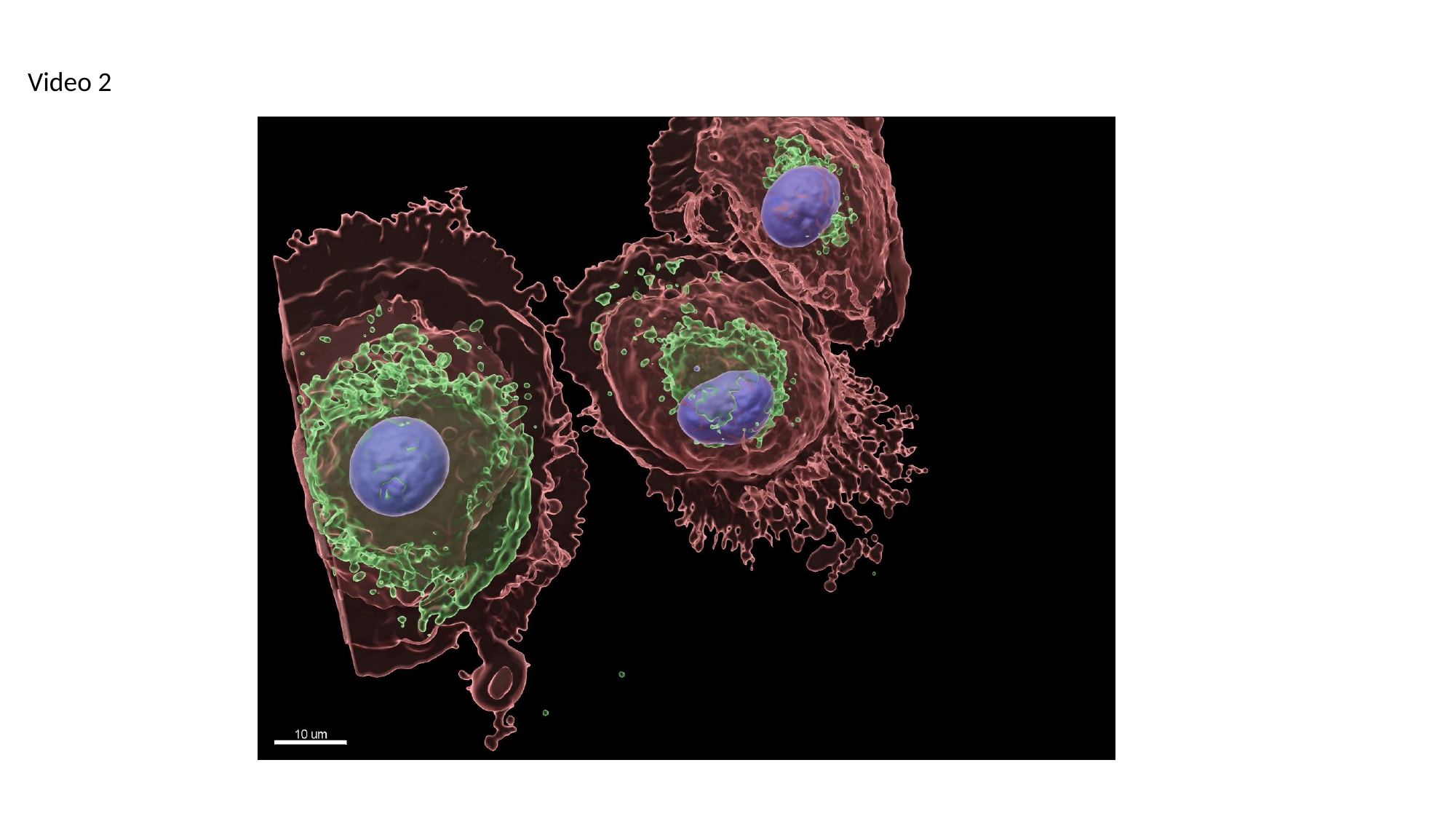

Video 2

Supplement: S1 Video — Human monocyte derived dendritic cells were co-incubated with B. malayi mf-derived EVs for 72 hours at 37°C in 5% CO2. The dendritic cells were labelled with PKH26 (red) and counterstained with VECTASHIELD Hardset Antifade Mounting Media with DAPI (blue) to visualize the nuclei. The parasite EVs were labelled with PKH67 (green). The video represents merged images allowing to visualize the different layers of cellular internalization. All images were captured using Zeiss 780. The assay was performed three times. (PPTX) [file pntd.0008884.s001.pptx]
